# Identification and characterization of hADSC‐derived exosome proteins from different isolation methods

**DOI:** 10.1111/jcmm.16775

**Published:** 2021-07-08

**Authors:** Lien‐Hung Huang, Cheng‐Shyuan Rau, Shao‐Chun Wu, Yi‐Chan Wu, Chia‐Jung Wu, Chia‐Wen Tsai, Chia‐Wei Lin, Tsu‐Hsiang Lu, Ching‐Hua Hsieh

**Affiliations:** ^1^ Department of Neurosurgery Kaohsiung Chang Gung Memorial Hospital and Chang Gung University College of Medicine Kaohsiung Taiwan; ^2^ Department of Anesthesiology Kaohsiung Chang Gung Memorial Hospital and Chang Gung University College of Medicine Kaohsiung Taiwan; ^3^ Department of Plastic Surgery Kaohsiung Chang Gung Memorial Hospital and Chang Gung University College of Medicine Kaohsiung Taiwan

**Keywords:** and iTRAQ, ExoQuick‐TC, ExoQuick‐TC ULTRA, exosomes, hADSC, size exclusion chromatography, ultracentrifugation

## Abstract

Exosomes are secreted into the extracellular space by most cell types and contain various molecular constituents, which play roles in many biological processes. Adipose‐derived mesenchymal stem cells (ADSCs) can differentiate into a variety of cell types and secrete a series of paracrine factors through exosomes. ADSC‐derived exosomes have shown diagnostic and therapeutic potential in many clinical diseases. The molecular components are critical for their mechanisms. Several methods have been developed for exosome purification, including ultracentrifugation, ultrafiltration, density gradient purification, size‐based isolation, polymer precipitation and immuno‐affinity purification. Thus, we employed four methods to isolate exosomes from the hADSC culture medium, including ultracentrifugation, size exclusion chromatography, ExoQuick‐TC precipitation and ExoQuick‐TC ULTRA isolation. Following exosome isolation, we performed quantitative proteomic analysis of the exosome proteins using isobaric tags for relative and absolute quantification (iTRAQ) labelling, combined with 2D‐LC‐MS/MS. There were 599 universal and 138 stably expressed proteins in hADSC‐derived exosomes. We proved that these proteins were potential hADSC‐derived exosomes markers, including CD109, CD166, HSPA4, TRAP1, RAB2A, RAB11B and RAB14. From the quantitative proteomic analysis, we demonstrated that hADSC‐derived exosome protein expression varied, with lipopolysaccharide (LPS) treatment, in the different isolation methods. Pathway analysis and proliferation, migration and endothelial tube formation assays showed varying effects in cells stimulated with hADSC‐derived exosomes from different isolation methods. Our study revealed that different isolation methods might introduce variations in the protein composition in exosomes, which reflects their effects on biological function. The pros and cons of these methods are important points to consider for downstream research applications.

## 1 INTRODUCTION

Exosomes are a discrete population of small extracellular vesicles (EVs), 30‐150 nm in size, and secreted into the extracellular space from most cell types.^1^ Exosomes represent a mode of intercellular communication though they contain various molecular constituents, including DNA, RNA, proteins and lipids.^2^, ^3^ Exosomes may play a role in immune response, signal transduction, antigen presentation, metabolism and cancer development.^4^, ^5^, ^6^, ^7^ Exosomes from lung spheroid cell could attenuate and resolve bleomycin‐ and silica‐induced fibrosis.^8^ Exosomes from bone marrow‐derived mesenchymal stem cells combine with atorvastatin pretreatment significantly improved cardiac function and promoted blood vessel formation.^9^

Adipose‐derived mesenchymal stem cells (ADSCs) are derived stromal cells originating from stromal‐vascular fragments of adipose tissue, with promising therapeutic potential.^10^ ADSCs can differentiate into a variety of cell types and secrete a series of paracrine factors that function in cell‐to‐cell communication, immunoregulation, angiogenesis, revascularization and tissue regeneration.^11^, ^12^, ^13^ Some paracrine factors are secreted through exosomes. Various studies have demonstrated that ADSC‐derived exosomes have diagnostic and therapeutic potentials in many clinical diseases.^14^, ^15^, ^16^, ^17^, ^18^

Lipopolysaccharide (LPS) derived from gram‐negative bacteria which stimulating immune cell activity and triggering the inflammatory response. Several studies showed that LPS stimulates growth factors production in mesenchymal stromal cells (MSCs).^19^ LPS enhanced survival of engrafted MSCs through VEGF expression and protect MSCs against apoptosis.^20^, ^21^ In rat model, LPS‐preconditioned MSCs transplantation can reduce apoptosis of myocardium and enhance cardiac function.^21^ LPS known to induce exosomes release and exosomes from LPS‐stimulated macrophages increase neuroprotection and functional improvement after ischaemic stroke.^22^, ^23^ ADSC paracrine levels of VEGF, FGF and EGF were also induced after LPS treatment and showed the therapeutic potential in acute lung injury.^24^ However, in current study the molecular function is still not understood.

The molecular components of ADSC‐derived exosomes are critical for their mechanisms of action. Exosome isolation and characterization are important for their application in biomedical sciences. However, there is no gold standard procedure for exosome purification. Several methods have been developed, including ultracentrifugation,^25^ ultrafiltration,^26^ density gradient purification,^2^ size‐based isolation,^27^ polymer precipitation and immuno‐affinity purification.^4^, ^28^, ^29^ The vesicle purity, yield and components may depend on the methods used. Thus, we analysed the protein components of hADSC‐derived exosomes, obtained using different isolation methods.

In this study, we isolated exosomes from the hADSC culture medium using four methods, including ultracentrifugation, size exclusion chromatography, ExoQuick‐TC precipitation and ExoQuick‐TC ULTRA isolation. Following isolation, we performed quantitative proteomic analysis of the exosome proteins using isobaric tags for relative and absolute quantification (iTRAQ) labelling, combined with 2D‐LC‐MS/MS. Using these analysis techniques, we investigated the protein components in hADSC‐derived exosomes, using different isolation methods.

## 2 MATERIALS AND METHODS

### 2.1 Cell culture and culture media collection

Human adipose‐derived stem cells (hADSC, PT‐5006, Clonetics, Lonza) were cultured in keratinocyte‐SFM (17005‐042, GIBCO‐Invitrogen), supplemented with 2 mmol/L N‐acetyl‐L‐cysteine (NAC, A8199, SIGMA), L‐ascorbic acid 2‐phosphate (Asc 2P, A8960, SIGMA) and 5% foetal bovine serum (16000044, GIBCO‐Invitrogen).

Human umbilical vein endothelial cells (HUVECs, BCRC No. H‐UV001) were cultured in medium 199, supplemented with 10% foetal bovine serum, 25 U/mL heparin (H‐3149, SIGMA), 30 µg/mL endothelial cell growth supplement (ECGS, 02‐102, Millipore), 2 mmol/L L‐glutamine, 1.5 g/L sodium bicarbonate and 1X penicillin/streptomycin.

For culture media collection, cell culture was limited to eight passages. 1 × 10^6^ hADSC cells were cultured in 10 mL serum‐free media, with/without 1 μg/mL lipopolysaccharides (LPS, L3755, SIGMA), for 24 hours. The culture media (500 mL) were harvested and centrifuged at 300 *g* for 5 minutes to remove cells and cell debris. The supernatants were concentrated using the Amicon^®^ Ultra‐15 (UFC900324, Merck‐Millipore) and transferred into new tubes for further use.

### 2.2 Exosome isolation

#### 2.2.1 Ultracentrifugation (UC)

Exosomes were separated from cell culture media via multiple centrifugation steps, as per the method described by Lin et al.^30^ Briefly, concentrated media were centrifuged at 2000 *g* for 20 minutes, then at 10 000 *g* for 30 minutes. The supernatant was harvested and centrifuged at 110 000 *g* for 60 minutes. The pellet was resuspended in phosphate‐buffered saline and stocked for further use.

#### 2.2.2 ExoQuick‐TC precipitation (TC)

Exosomes were purified from the cell culture media, using the ExoQuick‐TC^TM^ exosome precipitation solution (EXOTC50A‐1, system Biosciences), according to the manufacturer's instructions. Briefly, concentrated media were centrifuged at 3000 *g* for 15 minutes. The supernatant was transferred into a new tube, and equal volumes of the ExoQuick‐TC solution were added. After mixing, they were refrigerated at 4°C overnight, at least 12 hours, then centrifuged at 1500 *g* for 30 minutes. The supernatant was discarded, and pellet was resuspended in PBS and stocked for further use.

#### 2.2.3 ExoQuick‐TC ULTRA isolation (TCU)

Exosomes were purified from cell culture media using, the ExoQuick‐TC^®^ ULTRA EV isolation kit (EQULTRA‐20TC‐1, system Biosciences), according to the manufacturer's instructions. Briefly, the exosome pellet from the ExoQuick‐TC was resuspended in 200 μL buffer B and equal volumes of buffer A were added. After mixing, they were loaded into a prepared purification column and incubated at room temperature on a rotating shaker for 5 minutes. Exosomes were eluted by centrifuging at 1000 *g* for 30 seconds.

#### 2.2.4 qEV10 size exclusion column purification (qEV)

Exosomes were purified from the cell culture media using qEV10 size exclusion columns (iZON science), according to the manufacturer's instructions. Briefly, 10 mL of concentrated media was loaded into qEV size exclusion columns, followed by elution with phosphate‐buffered saline (PBS). Five millilitres of each fraction was collected and quantified, with a spectrophotometer (Tecan Sunrise™). Based on the exosomes size distribution and protein level, the fractions containing the vesicles were pooled and concentrated using the Amicon^®^ Ultra‐15 (UFC900324, Merck‐Millipore).

### 2.3 Nanoparticle tracking analysis (NTA)

Size determination and concentration measurements of hADSC exosomes were performed on NanoSight NS300 (Malvern Instruments, UK). All samples were diluted in PBS to a final volume of 1 mL. Following settings were set according to the manufacturer's software manual: camera used sCMOS mode and set camera level to level 16. For each measurement, set cell temperature at 25°C and set syringe pump speed to 70 µL/s. After capture, the videos were analysed using NanoSight Software NTA 3.4 Build 3.4.003 with a detection threshold of 5. Hardware: embedded laser: 45 mW at 488 nm; camera: sCMOS. The size distribution diagrams, mean/mode size values and standard deviations were calculated within the NTA 3.4 software.

### 2.4 Dynamic light scattering (DLS)

The size and zeta potential of exosomes was measured by dynamic light scattering coupled with a Zetasizer Nano ZS system (Malvern Instruments, UK). Briefly, exosomes samples from four methods were diluted to 1 mL of PBS and gently mixed to provide a homogeneous solution. The homogeneous solution was put in a disposable cuvette and transferred to a Malvern Clear Zeta Potential cell for the Zeta potential measurement. The data were analysed through Dispersion Technology Software V7.01 supplied by the Malvern Zetasizer Nano ZS. The mean particle diameter was calculated from the measured particle distributions, and polydispersity index (PdI) was given as a measure of the size ranges of the solution.

### 2.5 Transmission Electron Microscopy (TEM)

The bi‐lipid layer of exosomes was characterized with TEM, which was commissioned to MA‐tek at Hsin‐chu, Taiwan. Briefly, the isolated exosomes were fixed with 2% paraformaldehyde in 0.1 mol/L PBS at RT. After 15 minutes, place 5 μL of exosomes samples onto carbon‐coated 400 mesh Cu/Rh grids (Ted Pella Inc, Redding, CA) at RT for 1 minute. Blot the drop with filter paper and replace with a 5 μL drop of 1% uranyl acetate (Polysciences, Inc, Warrington, PA) in ddH_2_O for three times. The stained grids were examined with JEOL TEM‐2000 EX II microscope.

### 2.6 Exosome protein extraction and iTRAQ labelling

Exosome proteins were purified using the T‐PER tissue protein extraction reagent (78510, Thermo Scientific). The protein samples were desalted using the Amicon^®^ Ultra‐15 (Merck‐Millipore) and quantified using the BCA protein assay (23225, Thermo Scientific Pierce).

For iTRAQ labelling, 25 µg of the protein samples was dried using SpeedVac and resuspended in iTRAQ dissolution buffer [0.5 mol/L triethylammonium bicarbonate (TEAB), pH 8.5]. Protein samples were reduced, with the iTRAQ reduction buffer (tris‐2‐carboxyethyl phosphine, TCEP) at 60°C for 30 minutes, then alkylated in the dark, with iodoacetamide at 37°C for 30 minutes. After the protein samples were digested using sequencing grade modified trypsin (V511A, Promega), they were dried using SpeedVac. Next, the peptides were reconstituted in 10 µL iTRAQ dissolution buffer and mixed with 30 µL iTRAQ labelling reagents at RT for overnight (Applied Biosystems Inc, Foster City). iTRAQ‐labelled samples were dried using SpeedVac for further analysis.

### 2.7 2D LC‐MS/MS

The iTRAQ‐labelled samples were analysed using the Q ExactiveTM HF mass spectrometer (Thermo Fisher, San Jose), coupled with the Thermo Scientific™ UltiMate™ 3000 RSLCnano HPLC System, which was commissioned to the clinical proteomics core laboratory of Chang Gung Memorial Hospital at Linkou, Taiwan. Briefly, the iTRAQ‐labelled peptides were pooled and desalted using Sep‐Pak C18 cartridges (Waters). The desalted peptides were dried using SpeedVac and re‐suspended in 0.5% trifluoroacetic acid. The peptide mixtures were loaded onto a C18 column (EASY‐Spray™) and separated using 0.1% formic acid solution, with varying amounts of acetonitrile (5%‐80%). The top abundant fifteen precursor ions, within 375‐1400 m/z scan range, were dynamically selected for further fragmentation in high collision dissociation (HCD) mode, with normalized collision energy set to 33 ± 1. In full MS scan, the resolution was set to 60 000 at m/z 200, AGC target to 3e6 and maximum inject time to 50 ms In MS/MS scan, the resolution was set to 15 000, AGC target to 5e4 and maximum injection time to 100 ms The release of the dynamic exclusion of the selected precursor ions was set to 20 seconds.

### 2.8 Database search and protein quantification

The raw MS data were queried using the Mascot search algorithm (version 2.5, Matrix Science) against the Swiss‐Prot human protein database via Proteome Discoverer (version 2.1, Thermo Scientific) software. For protein identification, the search parameters were set as follows: carbamidomethylation at cysteine as the fixed modification, oxidation at methionine, acetylation at protein N‐terminus, iTRAQ‐labelled at peptide N‐terminus, lysine residue as dynamic modifications, 10 ppm and 0.02 Da for MS/MS tolerance and maximum missing cleavage sites with 2.

### 2.9 Antibodies

The commercially available primary antibodies used in this study included the following: monoclonal rabbit anti‐CD9 (92726, Abcam) and polyclonal rabbit anti‐Rab7 (2094, cell signalling). The secondary antibodies used for Western blotting included HRP‐conjugated goat anti‐rabbit IgG antibodies purchased from GE Healthcare.

### 2.10 WST‐1 proliferation assay

The CytoScan™ WST‐1 Cell Cytotoxicity Assay (11644807001, Roche) was used to measure HUVEC proliferation upon hADSC‐derived exosome treatment. Briefly, 2 × 10^4^ HUVECs were seeded in 96‐well plates per well, with 100‐µL culture media, for 24 hours. Exosomes (30 µg) from different isolation methods were added, then cultured for 24 hours. Finally, 10 µL of the WST‐1 Assay Dye Solution was added to each well and the plate incubated in the cell culture incubator overnight. Finally, the plates were measured at 450 nm using a microplate reader.

### 2.11 Wound healing cell migration assay

HUVECs (2 × 10^4^) were seeded in 2‐well silicone inserts (81176, ibidi) and incubated in the cell culture incubator overnight. Exosomes (30 µg) were added to each well, and the silicon inserts removed. Images were acquired using a light microscope. After 8 hours, images were acquired again. The migration areas were measured using the ImageJ software.

### 2.12 Endothelial tube formation assay

The Angiogenesis Assay (K905‐50, BioVision) was used to measure the tube forming ability of HUVEC upon hADSC‐derived exosome treatment. 96‐well plates were pre‐coated with extracellular matrix gel. HUVECs (1 × 10^4^) were seeded in the 96‐well and treated with 30 µg of exosomes, from different isolation methods. After 6 hours, the culture media were removed and the cells washed with wash buffer. The cells were stained, with staining dye at 37°C for 30 minutes. The images were acquired using a fluorescence microscope.

### 2.13 Statistical analyses

All data were processed using the GraphPad Prism 5 for Windows (version 5.01). Variables were presented as the mean ± standard deviation (SD). Pairwise comparisons were performed using the Student's t‐test and represented with a *P* value. All statistical tests were two‐tailed, and differences were considered significant at *P* < .05.

## 3 RESULTS

### 3.1 Exosomes isolation and identification

Exosomes were isolated from 500‐mL culture media. After different methods isolating, the exosome proteins yield was measured using absorbance at 280 nm and particles number was measured using NTA. The data showed that exosome proteins yield and particle number in UC group was higher than other groups (Figure S1).

The identification of exosomes from different isolated methods bases on size and morphology. The size distribution of exosomes was measured using DLS and NTA (Figure S2A,B), and the bi‐lipid layer morphology of exosomes was display by TEM (Figure S2C). The exosomes display a cup‐shaped, and the Z‐average of exosomes was 90.32 d.nm in UC group, 83.43 d.nm in qEV group, 69.3 d.nm in TC group and 83.38 d.nm in TCU group.

### 3.2 Generation of hADSC‐derived exosome proteomic data sets

**FIGURE 1:**
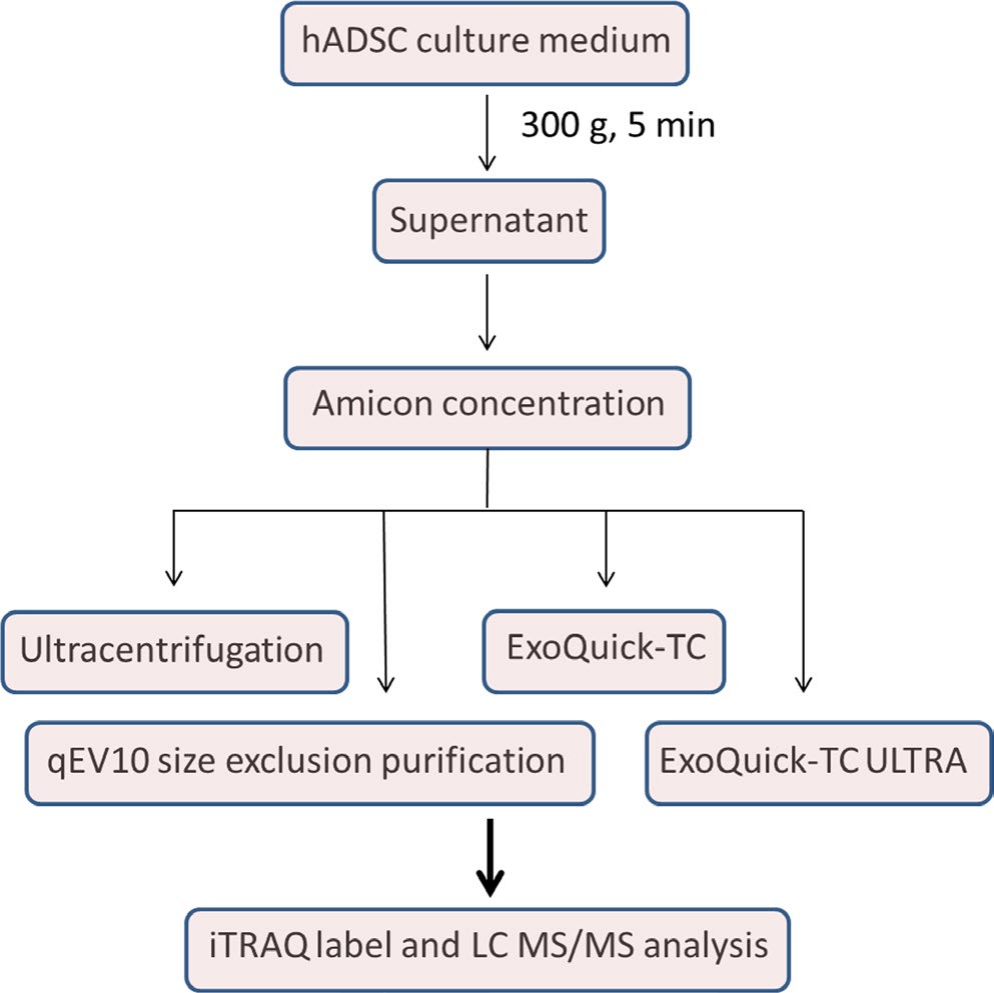
The experimental workflow used for exosome isolation

To identify the protein components in hADSC‐derived exosomes, we performed an iTRAQ‐based quantitative proteomic analysis to analyse differentially expressed proteins from different isolation methods, including ultracentrifugation, qEV‐10 size exclusion chromatography, ExoQuick‐TC precipitation and ExoQuick‐TC ULTRA isolation (Figure 1). hADSC were treated, with or without LPS, and the culture media harvested for further analyses. Exosomes purified by different isolation methods were labelled with 4‐plex iTRAQ reagents of varying masses (114‐117). The experimental design is summarized in Table S1. The exosomes from ultracentrifugation and qEV‐10 size exclusion chromatography were grouped (Group 1) and those from the ExoQuick‐TC precipitation and ExoQuick‐TC ULTRA isolation into another group (Group 2).

**FIGURE 2:**
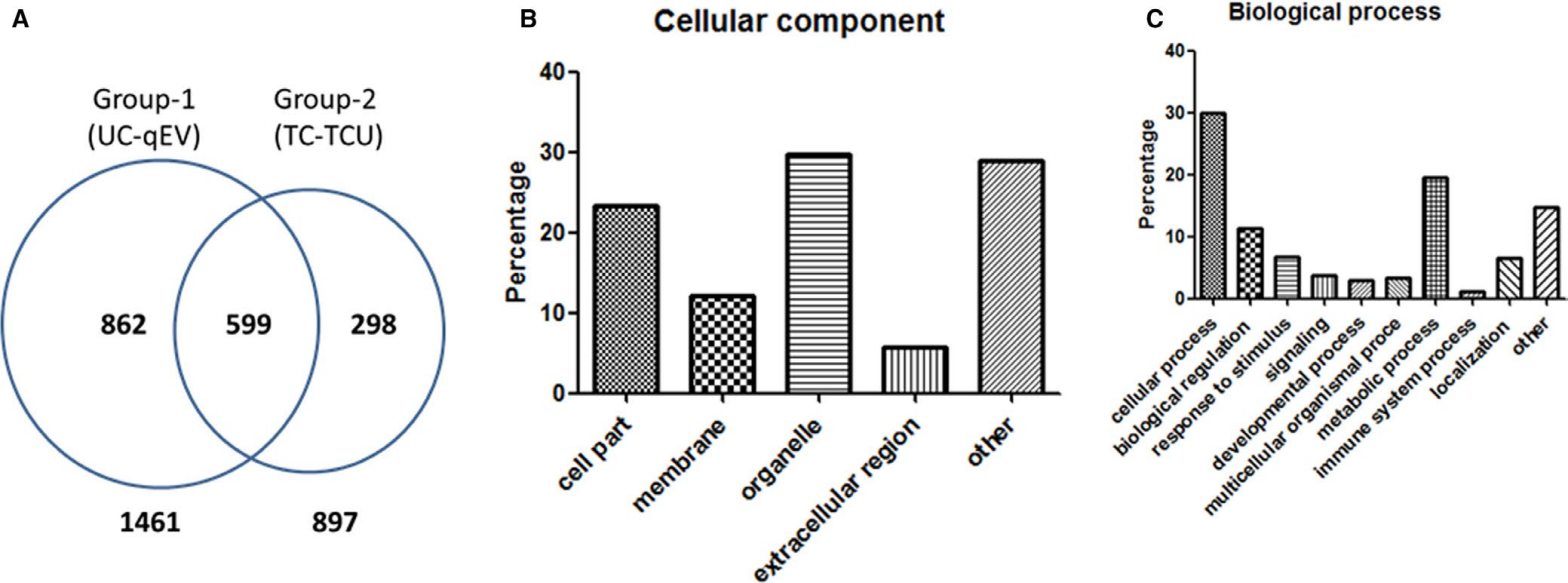
hADSC‐derived exosome protein identification. A, Venn diagram depicting the number of proteins common to the four isolation methods. Gene ontology analysis showing the cellular component (B) and biological processes (C) of the exosome proteins identified from the four isolation methods

We identified 1461 proteins and quantified 1136 proteins in Group 1 via 2D‐LC‐MS/MS analysis. In Group 2, we identified 897 proteins and quantified 801 proteins in Group 2 (Table S2). There were 599 exosome proteins from the four isolation methods, belonging to many protein families (Figure 2A, Table S3), including CD antigen (CD44, CD109 and CD166), heat shock proteins (HSPA1A, HSPA4, HSPA8, HSPB1, HAS90AA2P, HSP90AA1 and HSP90AB1), RAB proteins (RAB2A, RAB7A, RAB14 and RAB11B), proteasome proteins (PSMC6, PSMC1, PSMD12, PSMD2, PSMD3 and PSMD7), Annexins (ANXA 1‐6), ribosomal protein, etc CD9 and Alix are common exosome markers only found in Group 1; conversely, CD81 and TSG101 were only found in Group 2. We used Western blotting to verify the MS analysis results. The data showed that CD9 was detected in Group 1 (UC and qEV) but not in Group 2 (TC and TCU). Rab7 was detected in Group 1 and Group 2 (Figure S3). These results consist with MS analysis (Tables S2 and S3).

### 3.3 Characterization of hADSC‐derived exosome proteins

Using Gene Ontology analysis, we showed the cellular distribution of 599 hADSC‐derived exosome proteins (Figure 2B), which included 23.4% cell part, 12.1% membrane, 29.8% organelle and 5.7% extracellular region. A majority of the biological processes were cellular and metabolic processes, biological regulation, response to stimuli, localization and signalling (Figure 2C).

There were 138 proteins that were stably expressed in hADSC‐derived exosomes (Table 1). The fold change in protein expression was smaller than 1.5 in group 1 (UC, compare with qEV) and group 2 (TC, compare with TCU). These proteins, including CD109, CD166, HSPA4, TRAP1, RAB2A, RAB11B and RAB14, are potential biomarkers for hADSC‐derived exosome.

**TABLE 1:** Proteins list commonly involve in hADSC‐derived exosome

UniProt accession No.	Proteins	Exp. q‐value	Sequence coverage (%)	No. of Peptides	No. of PSMs	No. of Unique Peptides	MW [kD]	Abundance Ratio: UC/qEV (non)	Abundance Ratio: TC/TCU (non)
P31946	14‐3‐3 protein beta/alpha	0	45	11	37	4	28.1	1.251	0.725
P62258	14‐3‐3 protein epsilon	0	77	20	46	17	29.2	0.837	1.471
P27348	14‐3‐3 protein theta	0	58	14	44	8	27.7	0.709	1.018
Q4KWH8	1‐phosphatidylinositol 4,5‐bisphosphate phosphodiesterase eta‐1	0.001	0	1	3	1	189.1	0.954	1.028
O43242	26S proteasome non‐ATPase regulatory subunit 3	0	9	6	7	6	60.9	1.129	1.331
P25398	40S ribosomal protein S12	0	23	3	4	3	14.5	0.837	0.68
P08708	40S ribosomal protein S17	0	16	2	2	2	15.5	0.881	1.332
P15880	40S ribosomal protein S2	0	15	5	5	5	31.3	0.671	1.173
P21589	5'‐nucleotidase	0.001	2	1	1	1	63.3	1.13	1.241
P05387	60S acidic ribosomal protein P2	0	53	3	4	3	11.7	0.85	1.235
P62906	60S ribosomal protein L10a	0.003	4	1	1	1	24.8	0.691	0.716
P62913	60S ribosomal protein L11	0.002	4	1	1	1	20.2	0.732	1.195
P62829	60S ribosomal protein L23	0	19	3	3	3	14.9	0.783	1.08
Q9Y3U8	60S ribosomal protein L36	0.009	10	1	1	1	12.2	0.921	0.968
P11021	78 kD glucose‐regulated protein	0	50	35	96	32	72.3	1.399	0.982
O15144	Actin‐related protein 2/3 complex subunit 2	0	21	6	8	6	34.3	0.888	1.237
O15145	Actin‐related protein 2/3 complex subunit 3	0.001	6	1	2	1	20.5	1.132	1.268
P07741	Adenine phosphoribosyltransferase	0	16	3	5	3	19.6	1.143	0.761
P14550	Alcohol dehydrogenase [NADP(+)]	0	11	4	6	4	36.6	0.699	0.92
P05091	Aldehyde dehydrogenase, mitochondrial	0	24	8	13	6	56.3	0.687	1.088
P15121	Aldose reductase	0	18	3	4	3	35.8	0.678	0.963
Q9H4A4	Aminopeptidase B	0	10	6	7	6	72.5	1.416	1.297
P01008	Antithrombin‐III	0	6	3	4	3	52.6	1.091	0.865
P54136	Arginine‐‐tRNA ligase, cytoplasmic	0	3	2	2	2	75.3	0.741	1.057
P17174	Aspartate aminotransferase, cytoplasmic	0	12	5	11	5	46.2	0.9	1.003
P14868	Aspartate‐‐tRNA ligase, cytoplasmic	0	10	5	5	5	57.1	0.931	1.463
P53396	ATP‐citrate synthase	0	12	12	15	12	120.8	1.075	1.349
Q92499	ATP‐dependent RNA helicase DDX1	0	10	7	7	7	82.4	0.67	0.988
P07814	Bifunctional glutamate/proline‐‐tRNA ligase	0	2	3	3	3	170.5	1.359	1.14
P27824	Calnexin	0.006	2	1	1	1	67.5	1.361	1.372
P04632	Calpain small subunit 1	0.003	4	1	1	1	28.3	1.483	0.965
P17655	Calpain‐2 catalytic subunit	0	4	3	4	3	79.9	1.155	1.089
P00918	Carbonic anhydrase 2	0	8	2	4	2	29.2	0.868	1.042
Q6YHK3	CD109 antigen	0	3	4	4	4	161.6	1.058	0.704
Q13740	CD166 antigen	0	4	2	2	2	65.1	1.282	1.282
O00299	Chloride intracellular channel protein 1	0	30	6	7	6	26.9	0.925	0.733
O75390	Citrate synthase, mitochondrial	0	12	6	9	6	51.7	1.301	0.991
Q99715	Collagen alpha‐1(XII) chain	0	6	18	21	18	332.9	0.671	1.054
P12110	Collagen alpha‐2(VI) chain	0	24	20	35	20	108.5	0.696	1.07
P61201	COP9 signalosome complex subunit 2	0	16	6	8	6	51.6	0.837	1.02
Q9BT78	COP9 signalosome complex subunit 4	0	11	4	4	4	46.2	0.733	1.353
P60981	Destrin	0	49	9	14	8	18.5	0.989	0.833
Q14195	Dihydropyrimidinase‐related protein 3	0	29	11	20	9	61.9	0.863	0.82
P53634	Dipeptidyl peptidase 1	0	4	2	2	2	51.8	1.138	0.927
Q16531	DNA damage‐binding protein 1	0	14	17	23	17	126.9	1.432	0.974
Q13561	Dynactin subunit 2	0.001	6	2	2	2	44.2	1.313	1.17
O00429	Dynamin‐1‐like protein	0	4	3	3	3	81.8	0.929	1.158
Q12805	EGF‐containing fibulin‐like extracellular matrix protein 1	0.001	3	1	2	1	54.6	1.042	0.817
Q9NZN4	EH domain‐containing protein 2	0	5	3	3	2	61.1	0.769	0.907
P26641	Elongation factor 1‐gamma	0	11	6	9	6	50.1	0.805	0.775
Q9BS26	Endoplasmic reticulum resident protein 44	0	20	7	8	7	46.9	0.81	0.996
Q14240	Eukaryotic initiation factor 4A‐II	0	40	15	30	8	46.4	0.857	0.715
P62495	Eukaryotic peptide chain release factor subunit 1	0.009	3	1	1	1	49	0.787	0.943
P55884	Eukaryotic translation initiation factor 3 subunit B	0	9	5	7	5	92.4	0.791	1.189
Q9Y262	Eukaryotic translation initiation factor 3 subunit L	0	13	7	8	7	66.7	0.806	0.997
P47756	F‐actin‐capping protein subunit beta	0	8	2	2	2	31.3	0.963	0.727
Q16658	Fascin	0	14	6	8	6	54.5	0.771	0.775
P49327	Fatty acid synthase	0.001	0	1	1	1	273.3	0.919	1.49
P09972	Fructose‐bisphosphate aldolase C	0	35	13	22	9	39.4	1.171	1.043
P09104	Gamma‐enolase	0	34	10	16	7	47.2	0.789	0.759
Q9Y2G5	GDP‐fucose protein O‐fucosyltransferase 2	0.01	3	1	1	1	49.9	1.15	1.321
P06396	Gelsolin	0	14	11	14	11	85.6	1.158	0.773
P47897	Glutamine‐‐tRNA ligase	0.001	1	1	1	1	87.7	1.185	1.322
P62826	GTP‐binding nuclear protein Ran	0	25	6	10	6	24.4	0.719	0.8
P62879	Guanine nucleotide‐binding protein G(I)/G(S)/G(T) subunit beta‐2	0	19	6	7	3	37.3	1.094	1.414
P63244	Guanine nucleotide‐binding protein subunit beta‐2‐like 1	0	21	6	8	6	35.1	0.89	1.134
P34932	Heat shock 70 kD protein 4	0	25	16	20	15	94.3	0.892	0.798
Q12931	Heat shock protein 75 kD, mitochondrial	0	3	2	6	1	80.1	0.947	0.976
Q9Y4L1	Hypoxia up‐regulated protein 1	0	18	16	25	16	111.3	0.739	1.321
O00410	Importin‐5	0	7	8	9	8	123.6	0.868	0.799
P22692	Insulin‐like growth factor‐binding protein 4	0.001	12	2	2	2	27.9	0.852	1.229
P05556	Integrin beta‐1	0	7	6	6	6	88.4	1.06	1.335
Q9H0C8	Integrin‐linked kinase‐associated serine/threonine phosphatase 2C	0	7	2	2	2	42.9	0.749	0.774
O75874	Isocitrate dehydrogenase [NADP] cytoplasmic	0	21	10	13	10	46.6	0.975	0.995
P02788	Lactotransferrin	0	4	3	13	3	78.1	1.15	1.387
Q16363	Laminin subunit alpha‐4	0	3	5	5	5	202.4	0.85	1.277
P07942	Laminin subunit beta‐1	0	10	17	22	17	197.9	1.149	1.189
P11047	Laminin subunit gamma‐1	0	11	15	19	15	177.5	1.023	1.138
Q9NQ48	Leucine zipper transcription factor‐like protein 1	0	7	2	3	2	34.6	0.723	0.761
P10619	Lysosomal protective protein	0.001	2	1	1	1	54.4	0.831	1.01
Q9Y4K0	Lysyl oxidase homolog 2	0.001	2	2	2	2	86.7	0.895	0.897
Q9ULC4	Malignant T‐cell‐amplified sequence 1	0.001	6	1	1	1	20.5	1.18	1.18
Q9Y5P6	Mannose‐1‐phosphate guanyltransferase beta	0	21	6	9	6	39.8	0.683	0.844
P56192	Methionine‐‐tRNA ligase, cytoplasmic	0.001	1	1	1	1	101.1	0.752	1.013
P46821	Microtubule‐associated protein 1B	0	2	4	4	4	270.5	0.777	0.875
P28482	Mitogen‐activated protein kinase 1	0	19	7	10	5	41.4	1.137	0.984
O94760	N(G),N(G)‐dimethylarginine dimethylaminohydrolase 1	0	11	3	5	2	31.1	0.713	1.261
O95865	N(G),N(G)‐dimethylarginine dimethylaminohydrolase 2	0	8	2	3	1	29.6	1.041	0.726
P06748	Nucleophosmin	0	15	5	7	5	32.6	0.965	0.925
P19021	Peptidyl‐glycine alpha‐amidating monooxygenase	0.001	2	2	2	2	108.3	0.918	0.667
P62942	Peptidyl‐prolyl cis‐trans isomerase FKBP1A	0	25	2	3	2	11.9	1.173	1.441
Q15063	Periostin	0	28	19	26	19	93.3	0.764	0.931
Q06830	Peroxiredoxin‐1 OS=Homo sapiens	0	37	8	14	5	22.1	0.867	0.695
P30044	Peroxiredoxin‐5, mitochondrial	0	4	1	2	1	22.1	0.758	0.789
O95394	Phosphoacetylglucosamine mutase	0	8	4	5	4	59.8	1.087	1.224
P36955	Pigment epithelium‐derived factor	0	13	5	6	5	46.3	1	0.739
Q9UHX1	Poly(U)‐binding‐splicing factor PUF60	0	6	3	3	3	59.8	0.77	0.956
P0CG39	POTE ankyrin domain family member J	0	5	4	17	1	117.3	0.818	1.181
Q8N0Y7	Probable phosphoglycerate mutase 4	0	22	4	16	1	28.8	1.129	1.333
Q15113	Procollagen C‐endopeptidase enhancer 1	0	15	5	5	5	47.9	0.747	1.13
Q02809	Procollagen‐lysine,2‐oxoglutarate 5‐dioxygenase 1	0	5	4	4	4	83.5	0.848	1.048
P07737	Profilin‐1	0	28	4	5	4	15	0.801	0.944
Q15185	Prostaglandin E synthase 3	0	24	4	5	4	18.7	1.129	1.463
P25789	Proteasome subunit alpha type‐4	0	34	9	16	9	29.5	1.476	1.234
O14818	Proteasome subunit alpha type‐7	0	44	10	22	10	27.9	1.485	0.889
P28074	Proteasome subunit beta type‐5	0	34	9	14	9	28.5	0.984	0.866
O60502	Protein O‐GlcNAcase	0.002	1	1	1	1	102.8	0.918	0.95
P22061	Protein‐L‐isoaspartate(D‐aspartate) O‐methyltransferase	0	16	3	3	3	24.6	0.965	1.018
Q58FF6	Putative heat shock protein HSP 90‐beta 4	0	7	4	13	1	58.2	0.897	1.173
Q8NHP8	Putative phospholipase B‐like 2	0.008	1	1	1	1	65.4	0.946	1.232
Q15907	Ras‐related protein Rab‐11B	0	47	9	12	9	24.5	0.982	0.751
P61106	Ras‐related protein Rab‐14	0	58	11	19	10	23.9	1.171	1.244
P61019	Ras‐related protein Rab‐2A	0	33	7	11	7	23.5	1.038	0.777
Q15293	Reticulocalbin‐1	0.001	5	1	1	1	38.9	0.774	0.911
P00352	Retinal dehydrogenase 1	0	19	11	31	10	54.8	1.018	1.053
O94788	Retinal dehydrogenase 2	0	17	8	8	6	56.7	1.173	0.79
Q15257	Serine/threonine‐protein phosphatase 2A activator	0	8	3	4	3	40.6	0.839	0.967
P50454	Serpin H1	0.006	2	1	1	1	46.4	1.011	0.972
P42224	Signal transducer and activator of transcription 1‐alpha/beta	0	7	6	6	6	87.3	1.263	0.683
P05023	Sodium/potassium‐transporting ATPase subunit alpha‐1	0	13	11	12	11	112.8	1.476	1.374
P38646	Stress‐70 protein, mitochondrial	0	14	9	11	8	73.6	1.284	1.077
O00391	Sulfhydryl oxidase 1	0	6	4	5	4	82.5	0.731	0.734
P00441	Superoxide dismutase [Cu‐Zn]	0	22	3	3	3	15.9	1.002	1.333
Q9Y490	Talin‐1	0	13	28	29	24	269.6	1.353	1.362
Q08629	Testican‐1 OS=Homo sapiens	0.001	2	1	1	1	49.1	0.691	1.021
P52888	Thimet oligopeptidase	0	7	5	6	5	78.8	0.776	0.971
P35443	Thrombospondin‐4	0	3	2	3	1	105.8	1.441	0.812
P37837	Transaldolase	0	22	8	9	8	37.5	0.886	1.308
Q15582	Transforming growth factor‐beta‐induced protein ig‐h3	0	26	17	30	17	74.6	0.784	0.71
P54578	Ubiquitin carboxyl‐terminal hydrolase 14	0.001	2	1	1	1	56	0.736	0.775
Q93009	Ubiquitin carboxyl‐terminal hydrolase 7	0.002	1	1	1	1	128.2	0.994	1.057
P61086	Ubiquitin‐conjugating enzyme E2 K	0	14	2	2	2	22.4	0.761	0.832
P22314	Ubiquitin‐like modifier‐activating enzyme 1	0	17	15	17	15	117.8	0.96	0.691
A0AVT1	Ubiquitin‐like modifier‐activating enzyme 6	0	3	4	4	4	117.9	0.983	1.279
Q9NYU2	UDP‐glucose:glycoprotein glucosyltransferase 1	0.004	1	1	1	1	177.1	0.763	1.191
Q9Y224	UPF0568 protein C14orf166	0.009	3	1	1	1	28.1	1.486	1.073
Q16851	UTP‐‐glucose‐1‐phosphate uridylyltransferase	0	24	12	14	12	56.9	1.453	0.891
P21281	V‐type proton ATPase subunit B, brain isoform	0	23	9	10	9	56.5	1.21	0.992

### 3.4 Differences in the four kinds of isolation method

**FIGURE 3:**
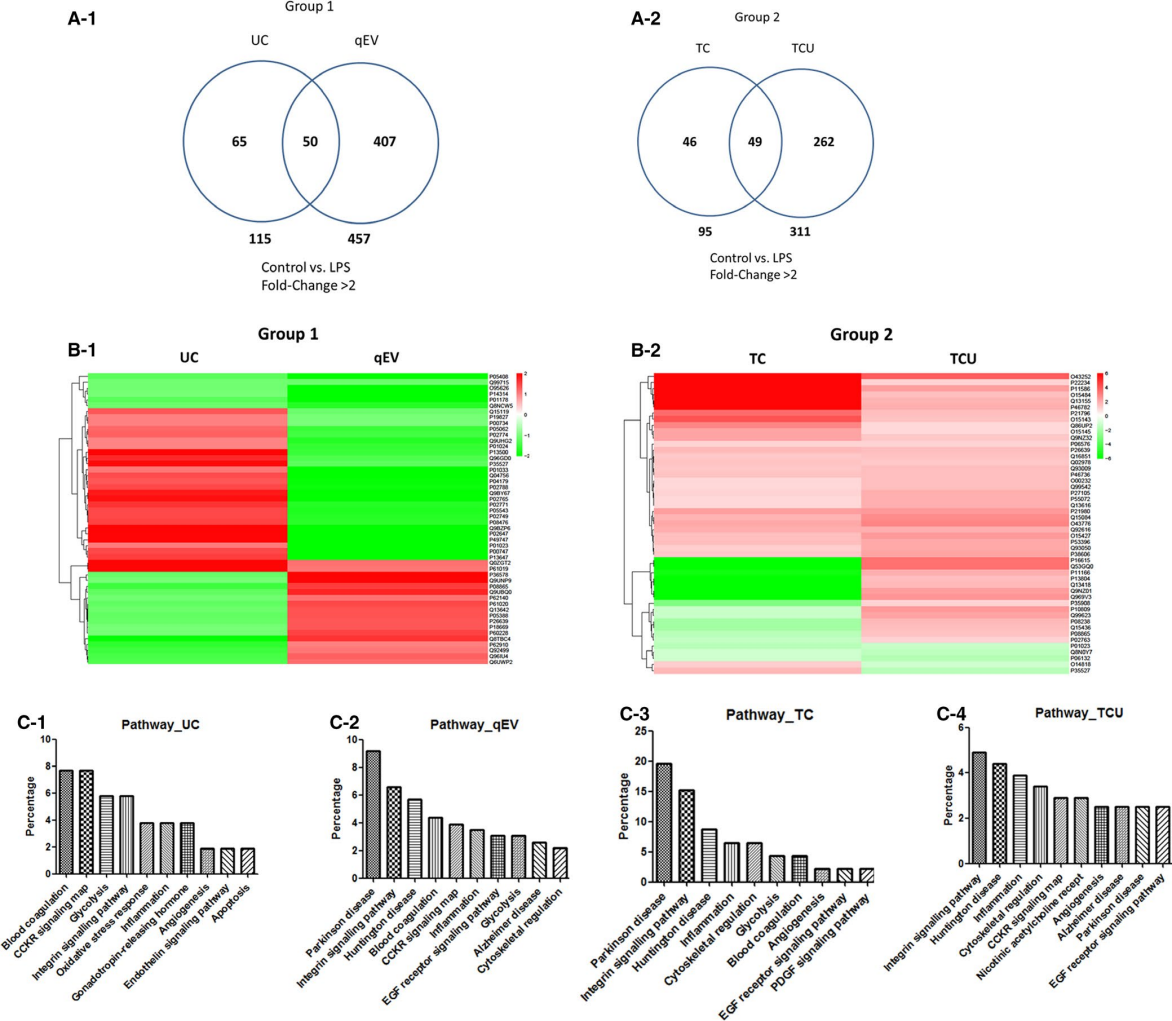
Hierarchical cluster analysis of differentially expressed proteins in the hADSC‐derived exosome. A, Proteins with a 2‐fold change in the four isolation methods were compared in each group. B, Hierarchical clustering of exosome proteome was performed via unsupervised hierarchical classification, and distance trees were constructed from differentially expressed proteins in each group. The isolation methods are shown in columns and proteins in rows. The heat map scale of fold‐change from −2 (green) to 2 (red). C, Pathway analysis of differentially expressed proteins in each group, based on universal Gene Ontology annotation terms

To investigate the differences in exosome component distribution from the four isolation methods, we analysed protein expression from LPS‐induced and normal hADSC‐derived exosome. The results demonstrated that there were 115 differentially expressed proteins, with twofold changes, from the UC isolation method (59 up‐ and 56 down‐regulated), 457 in the qEV isolation method (150 up‐ and 307 down‐regulated), 95 in the TC isolation method (55 up‐ and 40 down‐regulated) and 311 in the TCU isolation method (205 up‐ and 106 down‐regulated) (Figure 3A and Table S4). There is a difference in the number of exosome proteins regulated, after LPS treatment, in the four isolation methods.

Next, we found 50 differentially expressed proteins, with twofold changes in both UC and qEV, but with different expression profiles (Figure 3B, Table S5). In the TC and TCU groups, there were 49 proteins differentially expressed, with similar profiles. Surprisingly, there was almost no intersection of these two protein groups.

Through gene ontology analysis, we showed the pathways the differentially expressed proteins are involved in (Figure 3C). In all groups, these differentially expressed proteins were involved in the integrin signalling pathway and inflammation. In UC, these differentially expressed proteins were also involved in the cholecystokinin receptor (CCKR) signalling pathway, oxidative stress response and angiogenesis. In qEV, these proteins were involved in the CCKR signalling pathway, EGF receptor signalling pathway and cytoskeletal regulation. In TC, they were involved in cytoskeletal regulation, angiogenesis, and EGF and PDGF receptor signalling pathways. In TCU, they were involved in cytoskeletal regulation, angiogenesis and the EGF receptor signalling pathway.

Together, these results indicated that the different isolation methods affected the protein expression profiles after LPS treatment.

### 3.5 The biological function of LPS‐induced hADSC‐derived exosomes

**FIGURE 4:**
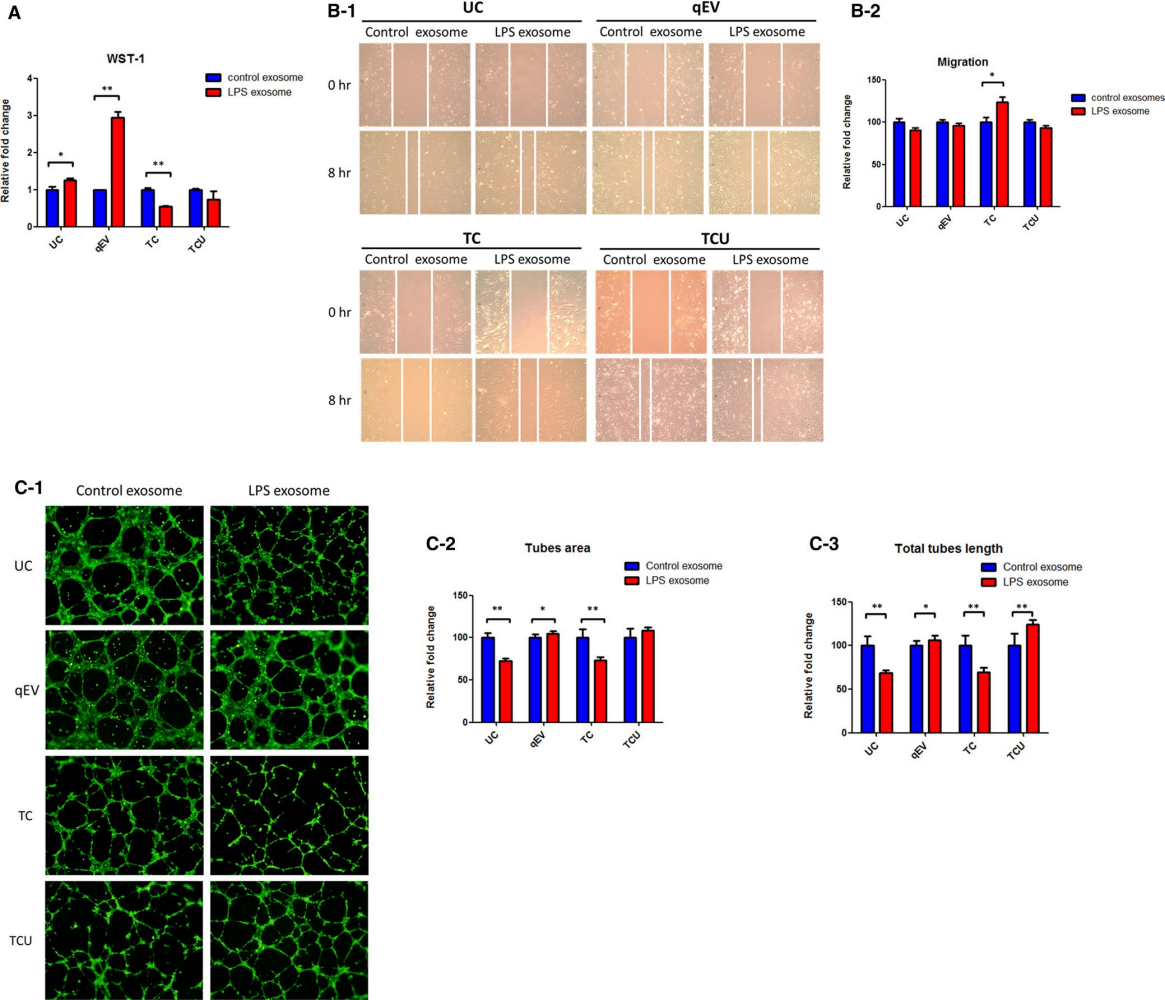
The effect of LPS‐induced exosomes. HUVECs were treated with hADSC‐derived exosomes, obtained with or without LPS treatment, followed by WST‐1 proliferation (A), migration (B) and endothelial tube formation (C) assays. The results are presented as the means ±SDs; * indicates significance *P* < .05, ** indicates significance *P* < .01, as assessed by the Student's t‐test

Pathway analysis, using WST‐1 proliferation, migration and endothelial tube formation assays, the biological function of LPS‐induced hADSC‐derived exosomes, obtained from the different isolation methods, was evaluated. In the WST‐1 proliferation assay, hADSC‐derived exosomes were added to HUVEC, with/without LPS treatment. LPS‐induced exosomes significantly increased cell proliferation in qEV and UC, but not in TCU (Figure 4A). However, LPS‐induced exosomes decreased cell proliferation in TC. In the wound healing migration assay, LPS‐induced exosomes did not affect the cell migration ability, except in TC (Figure 4B).

Next, we used the tube formation assay to evaluate angiogenesis. HUVECs were seeded in Matrigel‐coated 96‐well, and hADSC‐derived exosomes, with/without LPS, added. Through quantitative analysis of tube area and total tube length, LPS‐induced exosomes significantly decreased tube formation ability in UC and TC (Figure 4C). However, the tube formation ability of HUVECs increased after LPS‐induced exosome treatment in qEV. In TCU, LPS‐induced exosomes increased total tube length, but not tube area.

Together, these results indicated that LPS‐induced exosomes affected cell proliferation, migration and tube formation. However, the results were inconsistent with exosomes isolated using different methods.

## 4 DISCUSSION

In this study, we used ultracentrifugation, size exclusion chromatography, ExoQuick‐TC and ExoQuick‐TC ULTRA precipitation to isolate exosomes from hADSC culture medium. Quantitative proteomic analysis was performed to identify and quantify the protein content in the exosomes. We showed 599 proteins, which belonged to the same protein family, in all four isolation methods. CD antigen and heat shock proteins are commonly used exosome markers.^6^, ^31^, ^32^ Other protein families were also identified in the hADSC‐derived exosomes such as RAB, proteasome proteins, Annexins and ribosomal proteins (Table S3). Through iTRAQ labelling, we found 138 proteins that were stably expressed in hADSC‐derived exosomes, irrespective of the isolation method. These proteins are potential markers for hADSC‐derived exosomes, including CD109, CD166, HSPA4, TRAP1, RAB2A, RAB11B and RAB14. Although some proteins are commonly used as exosome markers such as CD9, CD81, Alix and TSG101, we found CD9 and Alix only in Group 1 (UC and qEV); conversely, CD81 and TSG101 were only found in Group 2 (TC and TCU). We do not rule out the effect of experimental limitations on this result. Contamination in mass spectrometry analyses is a major problem, which leads to ion suppression and interferes with protein identification.^33^, ^34^ One of the most common contaminants is polyethylene glycol (PEG) in samples.^35^ Exosomes were isolated via precipitation using polymers in TC and TCU. We could not eliminate these polymers in sample preparation and they interfere with protein identification.

The quantitative proteomic analysis demonstrated that the hADSC‐derived exosome protein expression and quantities varied, with LPS treatment and different isolation methods. For example, Rab5B was down‐regulated after LPS treatment in UC (LPS/control = 0.436), but up‐regulated in qEV (LPS/control = 2.833). Ultracentrifugation and size exclusion chromatography isolate exosomes based on different principles. It caused a dramatic change in the result of our experiment. This phenomenon was not observed in TC and TCU, which showed similar expression profiles. ExoQuick‐TC and ExoQuick‐TC ULTRA are the same series of products, with the same exosome isolation principle. Although similar proteins were purified with different methods, their proportions differed.

The different molecular contents of exosomes reflect their biological functions. Pathway analysis of differentially expressed proteins in each group showed diverse results. We also used cell proliferation, migration and endothelial tube formation assays to evaluate the biological functions of exosomes. These assays showed the varying effect of hADSC‐derived exosomes, from different isolation methods, on LPS‐stimulated cells. Exosome isolation methods may indirectly select for some vesicle subpopulations, with specific biochemical or physical characteristics, which affect the experimental outcome.^36^ This may explain the varying effects observed in the functional assay.

Exosomes are promising diagnostic, prognostic, therapeutic and drug delivery tools in clinical settings. Uniformity and quality are major challenges associated with exosome application. Many studies revealed the efficiency, yield and purity, size‐distribution, RNA/protein quality and miRNA composition of isolated exosomes.^28^, ^37^, ^38^ These reports revealed that different isolation methods introduced variations in exosomal component distribution. Each method has certain benefits and drawbacks such as purity, isolation scale and desired ease for a particular application. Through our quantitative proteomic analysis, we provided a set of hADSC‐derived exosomal marker proteins, which were independent of the isolation method. It may be beneficial to identify exosomes and develop affinity chromatography for their isolation.

## 5 CONCLUSION

In conclusion, our study revealed that different isolation methods might introduce diversity in the protein composition of exosomes, which reflects their various effects on biological function. We focused on the protein composition in this study. The DNA, RNA and lipid contents and amount varied, with methodological differences. The pros and cons of these methods are important points to consider for downstream research applications.

## CONFLICT OF INTEREST

The authors declare that they have no competing interests.

## AUTHOR CONTRIBUTIONS

**Lien‐Hung Huang:** Writing‐original draft (lead); Writing‐review & editing (lead). **Cheng‐Shyuan Rau:** Writing‐review & editing (supporting). **Shao‐Chun Wu:** Methodology (equal). **Yi‐Chan Wu:** Methodology (equal). **Chia‐Jung Wu:** Software (equal). **Chia‐Wen Tsai:** Software (equal). **Chia‐Wei Lin:** Visualization (lead). **Tsu‐Hsiang Lu:** Validation (lead). **Ching‐Hua Hsieh:** Conceptualization (lead); Funding acquisition (lead).

## Supporting information

Fig S1‐S3

Fig S2

Fig S3

Table S1

Table S2

Table S3

Table S4

Table S5
