# Supplementary material for: Identification and characterization of hADSC‐derived exosome proteins from different isolation methods
Source: J Cell Mol Med. 2021 Jul 8;25(15):7436–50. doi: 10.1111/jcmm.16775 (PMC8335681; doi:10.1111/jcmm.16775)
Supplement: Supplementary file 1 — Fig S1‐S3 [file JCMM-25-7436-s007.docx]

**Supplementary Figures**


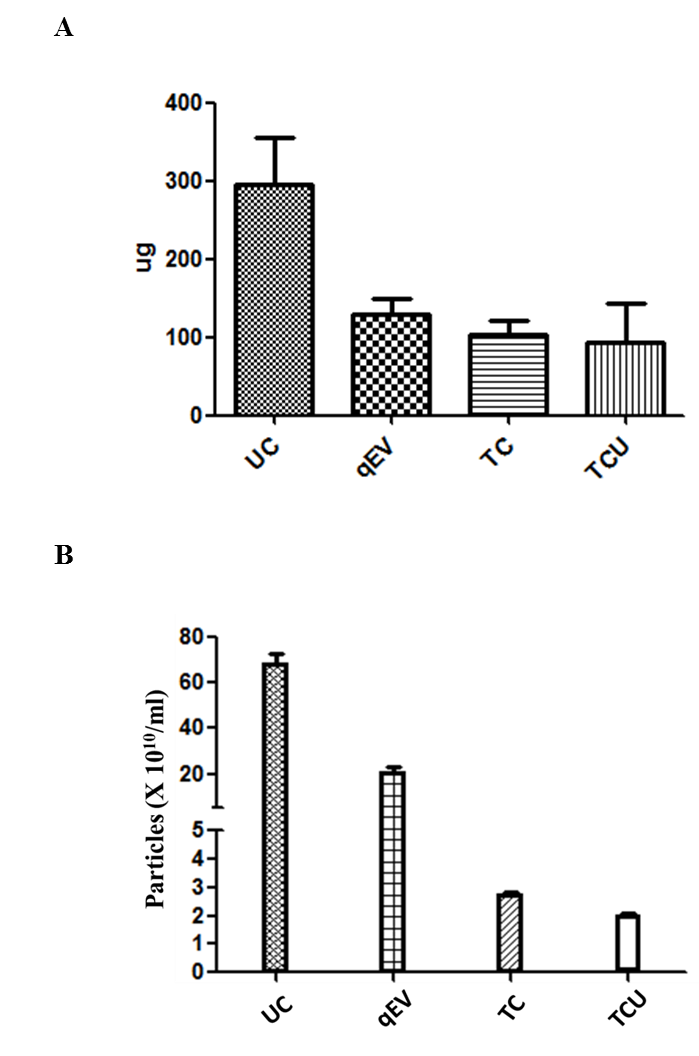


**Supplementary Figure S1.** **Comparison of various methodologies for exosome proteins isolation.** Exosomes were isolated from 500 ml culture media. After different methods isolating, (A) the exosomes proteins in UC, qEV, TC and TCU group were estimated by BCA protein assay. The results are presented as the means ± SEM. (B) the particle numbers of exosomes were measured using NTA. The results are presented as the means ± SD.


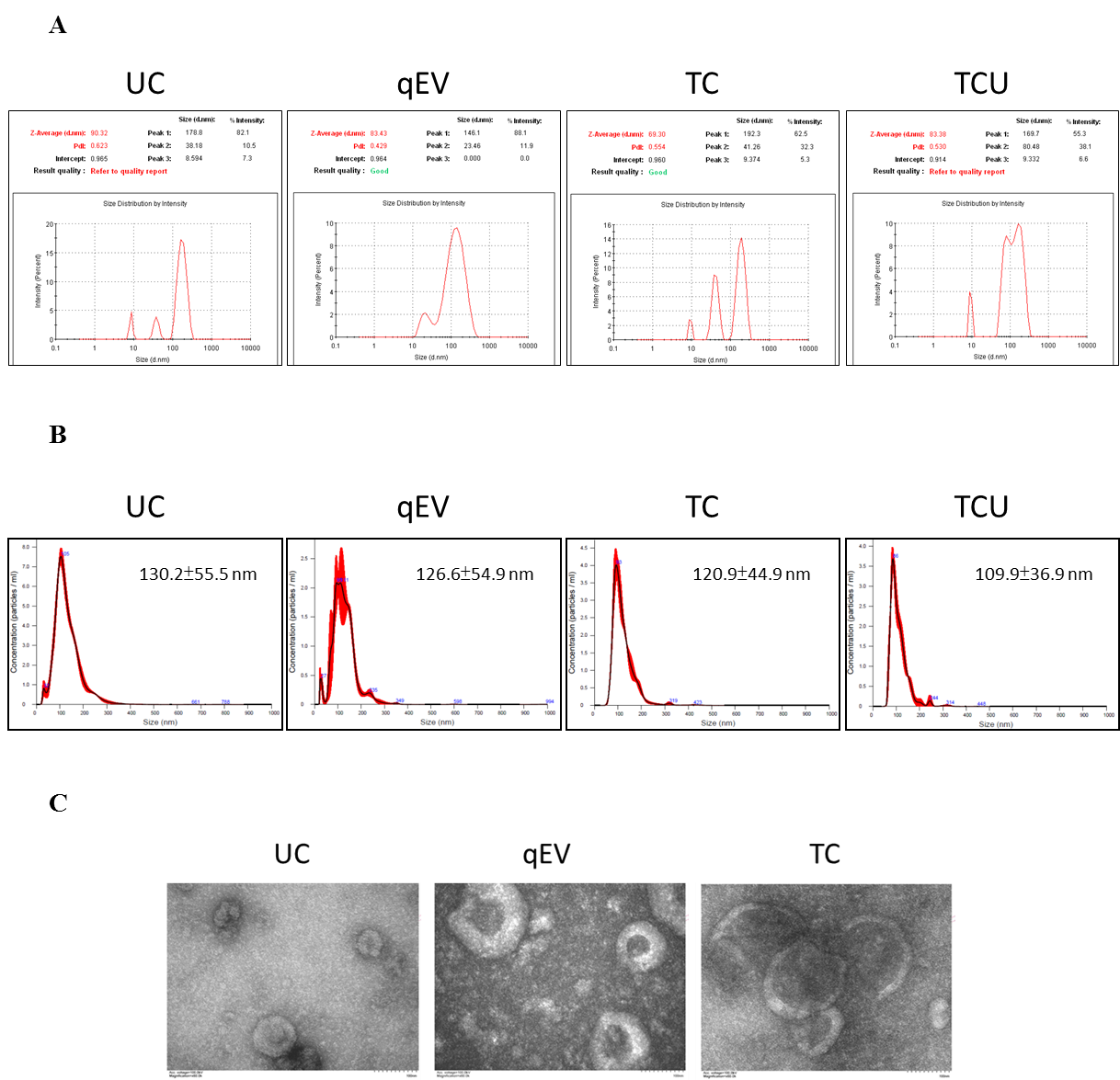


**Supplementary Figure S2. The exosome size distribution and structure.** Size distribution of exosomes were analyzed using DLS (A) and NTA (B). The morphology of exosomes were displayed by TEM images (C).


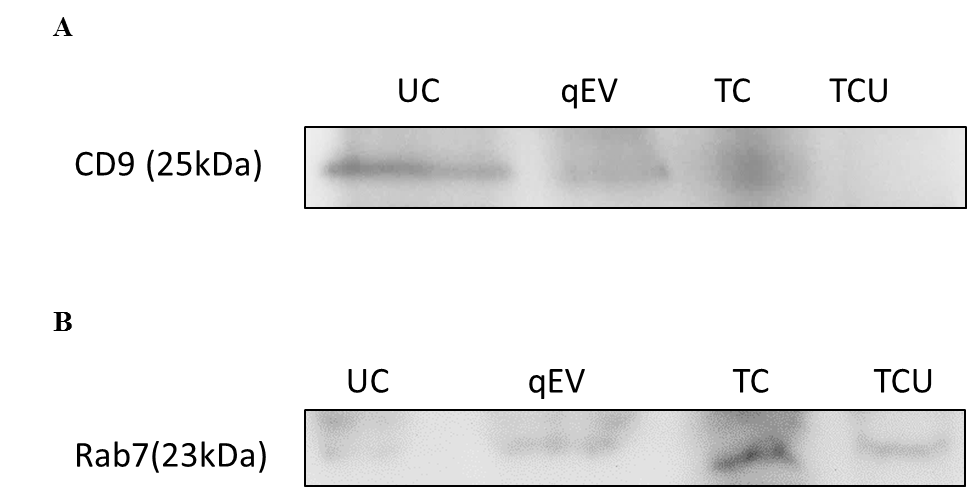


**Supplementary Figure S3. Exosomes proteins expression verify by western blotting.** 30ug exosomes proteins were resolved on a 12.5% SDS PAGE gel and analyzed by western blotting using an anti-CD9 antibody and anti-Rab7 antibody.
