# Supplementary figures and images for: Identification and characterization of hADSC‐derived exosome proteins from different isolation methods
Source: J Cell Mol Med. 2021 Jul 8;25(15):7436–50. doi: 10.1111/jcmm.16775 (PMC8335681; doi:10.1111/jcmm.16775)

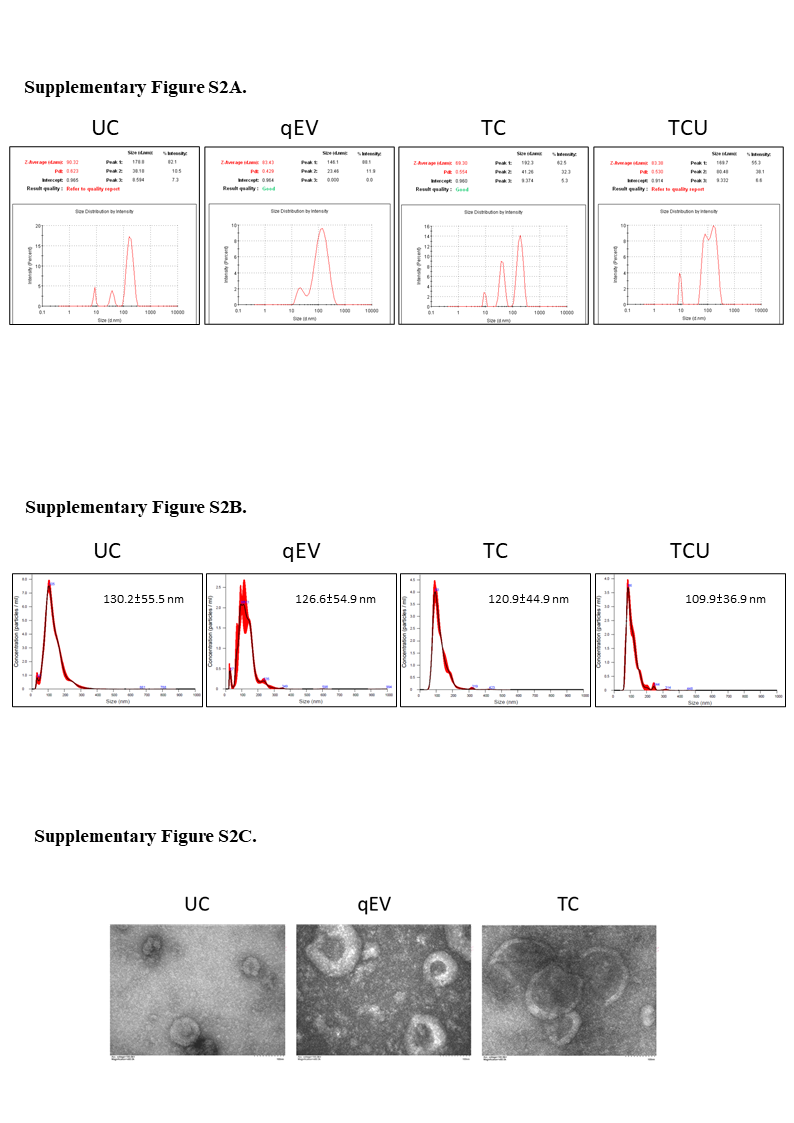

Supplement: Supplementary file 2 — Fig S2 [file JCMM-25-7436-s006.tif]

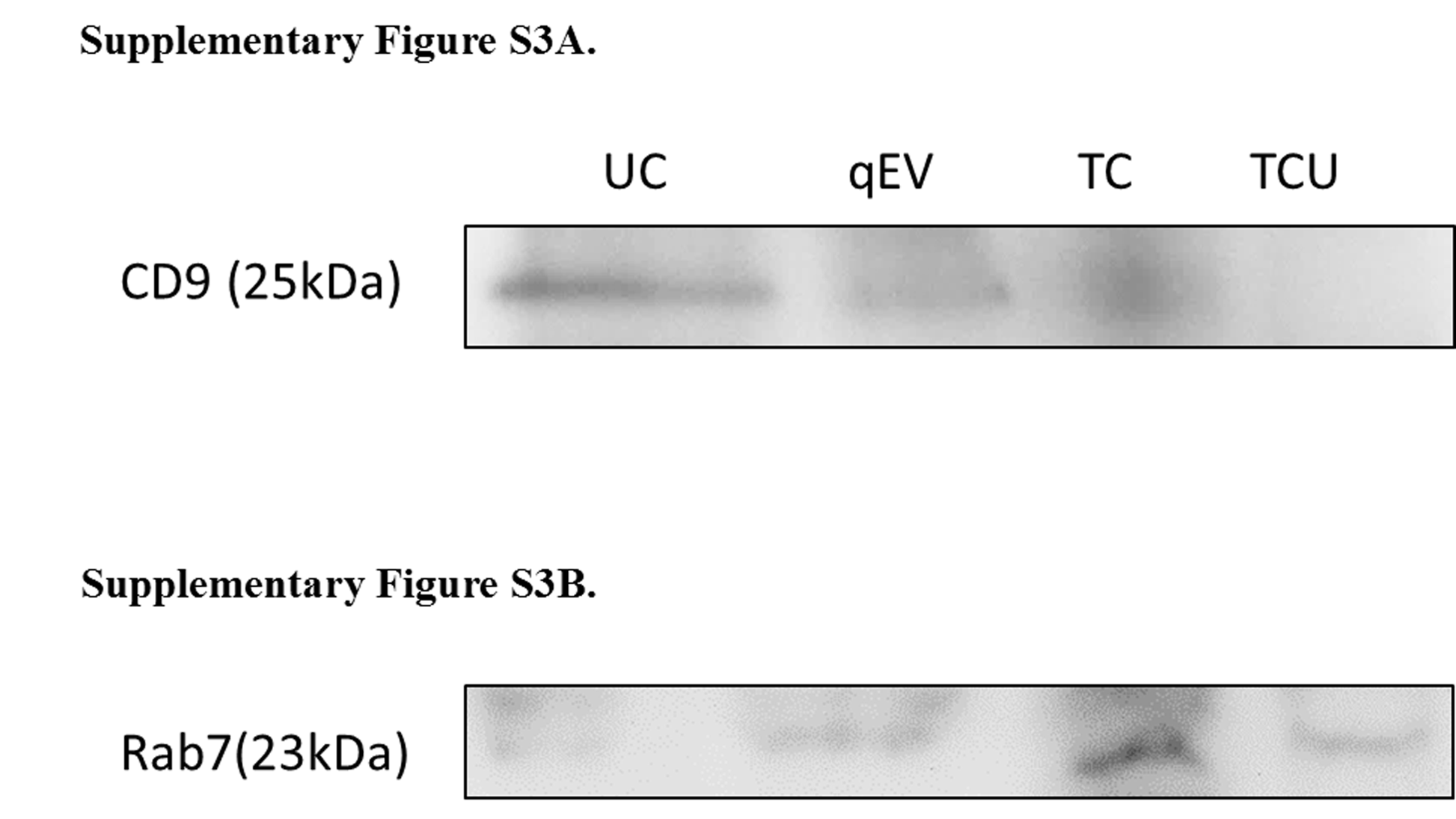

Supplement: Supplementary file 3 — Fig S3 [file JCMM-25-7436-s005.tif]
